# Regional heterogeneity in the membrane properties of mouse striatal neurons

**DOI:** 10.3389/fncel.2024.1412897

**Published:** 2024-07-31

**Authors:** Nao Chuhma, Stephen Rayport

**Affiliations:** ^1^Department of Molecular Therapeutics, New York State Psychiatric Institute, New York, NY, United States; ^2^Department of Psychiatry, Columbia University, New York, NY, United States

**Keywords:** membrane properties, excitability, spiny projection neurons, cholinergic interneurons, fast-spiking interneurons, striatum, multivariate analysis

## Abstract

The cytoarchitecture of the striatum is remarkably homogeneous, in contrast to the regional variation in striatal functions. Whether differences in the intrinsic membrane properties of striatal neurons contribute to regional heterogeneity has not been addressed systematically. We made recordings throughout the young adult mouse striatum under identical conditions, with synaptic input blocked, from four major striatal neuron types, namely, the two subtypes of spiny projection neurons (SPNs), cholinergic interneurons (ChIs), and fast-spiking GABAergic interneurons (FSIs), sampling at least 100 cells per cell type. Regional variation manifested across all cell types. All cell types in the nucleus accumbens (NAc) shell had higher input impedance and increased excitability. Cells in the NAc core were differentiated from the caudate-putamen (CPu) for both SPN subtypes by smaller action potentials and increased excitability. Similarity between the two SPN subtypes showed regional variation, differing more in the NAc than in the CPu. So, in the Str, both the intrinsic properties of interneurons and projection neurons are regionally heterogeneous, with the greatest difference between the NAc and CPu; greater excitability of NAc shell neurons may make the region more susceptible to activity-dependent plasticity.

## 1 Introduction

The striatum (Str) receives extensive cortical, thalamic, and ventral midbrain dopamine neuron input and mediates functions extending from reward processing to motivation, decision-making, and motor control (Wickens et al., 2007; Liljeholm and O’Doherty, 2012; Basile et al., 2021). Despite regional differences in mediated functions, the Str has a remarkably homogeneous cytoarchitecture (Gerfen and Bolam, 2016), with over 95% GABAergic spiny projection neurons (SPNs). There are two subtypes of SPNs, the direct-pathway SPNs (dSPNs), which express D1 dopamine receptors (D1R) and project directly to the ventral midbrain, and indirect-pathway SPNs (iSPNs), which express D2 dopamine receptors (D2R) and project via the pallidum. The remaining 5% of Str neurons comprise interneurons: cholinergic interneurons (ChIs) and several classes of GABAergic interneurons, notably the well-studied fast-spiking interneurons (FSIs; Tepper et al., 2018). Despite their small numbers, interneurons exert significant control over local circuit function (Tanimura et al., 2018; Tepper et al., 2018; Abudukeyoumu et al., 2019; Nahar et al., 2021).

Brain slice studies have revealed that Str neurons have distinct membrane properties. For instance, cholinergic interneurons (ChIs) are spontaneously active and respond to negative current injection with a voltage sag, while SPNs are silent and show no voltage sag. These distinct membrane properties play a crucial role in the computation of input signals and information processing within the Str, potentially showing regional heterogeneity. While these properties have been studied extensively in the dorsal striatum, particularly the anterior half of the caudate-putamen (CPu) in rodents (Basile et al., 2021), recent attention has extended to the posterior Str, as a mediator of behavioral functions distinct from those of the anterior CPu, with differing input–output connectivities and distributions of dSPNs and iSPNs (Menegas et al., 2015, 2017; Miyamoto et al., 2019; Valjent and Gangarossa, 2021). Information on the excitability of neurons in the posterior CPu remains limited, making it uncertain whether observations of cellular properties in the anterior CPu can be extended to the posterior part of the CPu. While the membrane properties of neurons in the CPu and the nucleus accumbens (NAc) likely differ, previous studies have focused more on subregional differences within the NAc (Maria-Rios et al., 2023). Despite many reports of Str neuron membrane properties in rodents, variations in species, animal age, locations of recordings, and recording methodologies across the different studies impede meta-analysis.

To assess regional variation in intrinsic membrane properties of Str neurons in the young adult mouse systematically, we recorded from four identified cell types under identical recording conditions, spanning the anterior–posterior extent of the Str, sampling a minimum of 100 cells of each cell type, with synaptic input blocked pharmacologically. We measured a consistent set of parameters for each cell and addressed regional variation in a multivariate analysis. These data were obtained in the course of mapping dopamine neuron synaptic input to identified Str neurons (Chuhma et al., 2023) in the same DAT-IREScre; Ai32 strain used for the synaptic mapping.

## 2 Materials and methods

### 2.1 Mice

Mice were handled in accordance with the guidelines of the National Institutes of Health Guide for the Care and Use of Laboratory Animals under the protocol NYSPI-1494 approved by the Institutional Animal Care and Use Committee of New York State Psychiatric Institute. Mice were group housed and maintained on a 12-h light/dark cycle. Food and water were supplied *ad libitum*. All slice/tissue preparations were performed during the light phase. A total of 295 mice (140 male and 155 female) of 2–3 months of age (postnatal days 60–93) were used. We balanced sampling from males and females for each dataset and location to avoid sex bias as possible. Since no apparent differences were recognized in cell properties obtained from males and females, the data were combined. For distribution of basic membrane parameters by sex and cell type, see Supplementary Table S1.

Mice were of C57BL6J/129Sv mixed background, backcrossed more than five times to C57BL6J, and kept inbred. D2-EGFP mice, originally on a FVB background, were crossed to C57BL6J more than eight times. DAT (Slc6a3)-internal ribosome entry site (IRES) cre (DAT^IREScre^) mice (Bäckman et al., 2006; Jackson Laboratories, Bar Harbor, ME; RRID:IMSR_JAX:006660) were mated with ROSA26-floxSTOP-CAG-ChR2-EYFP (Ai32; ChR2-EYFP; RRID:IMSR_JAX:024109). Most of the recordings were obtained from the same cells previously reported in our study of dopamine neuron synaptic transmission (393/432 cells; Chuhma et al., 2023), although membrane properties other than resting membrane potentials were not previously reported. Since the dataset for this study was obtained mostly during the course of our dopamine neuron synaptic mapping study (Chuhma et al., 2023), all the data were obtained from DAT^IREScre^; Ai32 mice, although this strain was not required for the present study. For the identification of dSPNs, iSPNs, and FSIs, mice with fluorescent genetic markers for each neuron type, D1-tdTomato (RRID:IMSR_JAX:016204), D2-EGFP (GENSAT; RRID:MMRRC_000230-UNC), or PV-tdTomato (RRID:IMSR_JAX:027395), respectively, were mated with DAT^IREScre^; Ai32 double mutant mice. For recording from ChIs, double mutant DAT^IREScre^; Ai32 mice without post-synaptic cellular markers were used, and ChIs were identified by their large soma size, which was confirmed by their distinctive membrane properties (Chuhma et al., 2014).

### 2.2 Brain slice electrophysiology

Mice were anesthetized with ketamine (90 mg/kg)/xylazine (7 mg/kg). After the confirmation of deep anesthesia, mice were decapitated, and the brains were quickly removed in ice-cold high-glucose artificial cerebrospinal fluid (ACSF; in mM: 75 NaCl, 2.5 KCl, 26 NaHCO_3_, 1.25 NaH_2_PO_4_, 0.7 CaCl_2_, 2 MgCl_2_ and 100 glucose, pH 7.4) and saturated with carbogen (95% O_2_ and 5% CO_2_). Coronal 300 μm Str sections were cut with a vibrating microtome (VT1200S, Leica, Buffalo Grove, IL), allowed to recover in high-glucose ACSF at room temperature for at least 1 h, transferred to the recording chamber (submerged, 0.5 mL of volume) on the stage of an upright microscope (BX61WI, Olympus, Tokyo, Japan), continuously perfused with standard ACSF (in mM: 125 NaCl, 2.5 KCl, 25 NaHCO_3_, 1.25 NaH_2_PO_4_, 2 CaCl_2_, 1 MgCl_2_ and 25 glucose, pH 7.4) and saturated with carbogen at 31–33°C.

D2-EGFP expression was confirmed by 470 nm LED field illumination; D1-tdTomato and PV-tdTomato expression was confirmed by 530 nm LED illumination (DC4100, Thorlabs, Newton, NJ). Recorded neurons were visualized using enhanced visible light differential interference contrast (DIC) optics with a scientific c-MOS camera (ORCA-Flash4.0LT, Hamamatsu Photonics, Hamamatsu, Japan). ChIs were identified visually by larger soma size and confirmed by spontaneous firing, shallow resting membrane potentials (approximately −60 mV), and voltage sag with −400 pA current injection (duration of 700 ms; Chuhma et al., 2014). Cells with spontaneous burst firing were discarded because they could be spontaneously active bursty GABAergic interneurons (Tepper et al., 2018). Genetic markers (ChAT-EGFP) were not used for the identification of ChIs because ChAT-EGFP mice overexpress vesicular acetylcholine transporter (VAChT; Nagy and Aubert, 2012; Crittenden et al., 2014), which may affect intrinsic membrane properties. Recording patch pipettes (3–7 MΩ) were fabricated from standard-wall borosilicate glass capillary with filament (World Precision Instruments). Intracellular solution was (in mM): 135 K^+^-methane sulfonate (MeSO_4_), 5 KCl, 2 MgCl_2_, 0.1 CaCl_2_, 10 HEPES, 1 EGTA, 2 ATP, and 0.1 GTP, pH 7.25. All recordings were conducted under whole cell current clamp with an Axopatch 200B amplifier (Molecular Devices, San Jose, CA; RRID:SCR_018866) or an Integrated Patch Amplifier (IPA; Sutter Instrument, Novato CA). Step currents, which were 700 ms in duration and amplitudes stepped from −400 pA to 300 pA with 50 pA steps, were injected to obtain the input–output curve. When cells were not excited by 300 pA current injection, step current amplitudes were increased up to 700 pA (in 50 pA steps) to obtain at least two different current injection steps with firing. Series resistance (8–30 MΩ) was compensated online by 70–80%. Liquid junction potentials (~10 mV) were adjusted online.

To avoid potentially regionally heterogeneous effects of spontaneous synaptic effects, glutamate, GABA, and acetylcholine (ACh) receptors were continuously blocked during recording. Antagonists and their concentrations were: iGluR AMPA/kainate CNQX 20 μM, iGluR NMDA D-APV 50 μM, mGluR1 JNJ16259685 5 μM, GABA_A_R SR95531 (gabazine) 10 μM, GABA_B_R CGP55845 3 μM, nAChR mecamylamine 10 μM, and mAChR scopolamine 2 μM. Stock solutions of drugs were prepared in either water or DMSO and diluted 1,000 to 5,000-fold in recording ACSF. Drugs were applied by perfusion. The antagonist-isolation cocktail was applied at least 10 min before recording. A maximum of 10 cells were recorded per animal.

**Figure 1:**
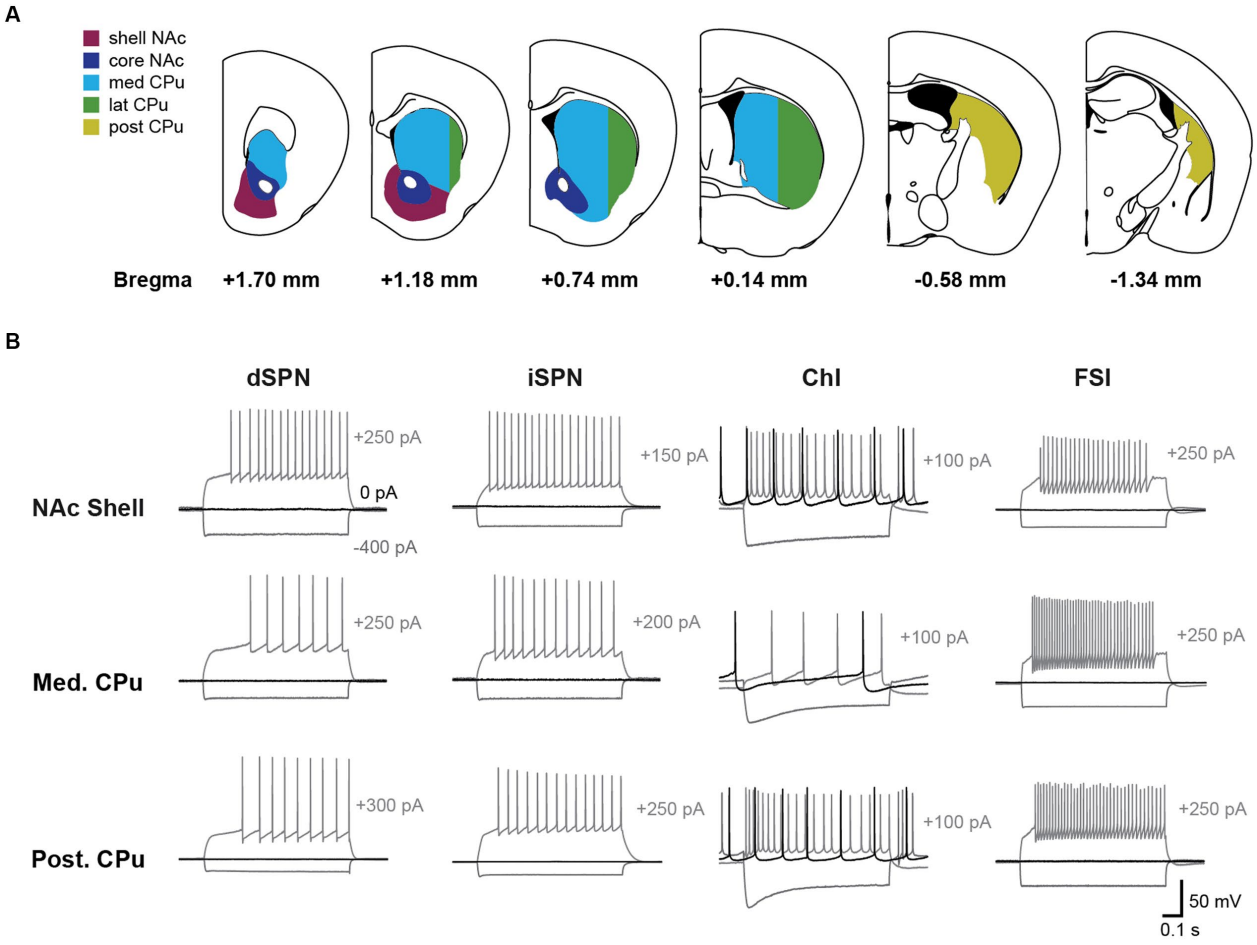
Firing patterns of striatal neurons vary by cell-type and subregion. **(A)** Recordings were made across five Str subregions; NAc shell (magenta), NAc core (dark blue), medial CPu (cyan), lateral CPu (green), and posterior CPu (dark yellow). Subregions are delineated in coronal sections and referenced to bregma. The CPu was split at −0.5 mm from bregma to delineate the anterior and posterior CPu; the anterior CPu was further split at 2 mm lateral to delineate the medial and lateral CPu. **(B)** Voltage recordings with step current injections are shown for dSPNs, iSPNs, ChIs, and FSIs in three subregions. Three traces are shown for each cell, with hyperpolarizing current injection (−400 pA; gray), no current injection (0 pA; black), or depolarizing current injection (gray; magnitude indicated to the right of the traces).

Data were filtered at 10 kHz with a 4-pole Bessel filter, digitized at 10 kHz (Digidata 1550A, Molecular Devices, or IPA, Sutter Instrument), and recorded using pClamp 10 (Molecular Devices; RRID:SCR_011323) or Sutter Patch 2.1.0 (Sutter Instrument) on Igor Pro 8.04 (Wavemetrics; RRID:SCR_000325). Prior to recording, landmarks (e.g., ventricle shape, location and shape of anterior commissure, and corpus callosum) were checked and slices with extreme tilt were discarded. If slices were mildly tilted, recordings were made in identifiable locations proximate to landmarks. Locations of recorded cells were manually mapped on mouse atlas (Paxinos and Franklin, 2008) coronal sections, on a 50-μm medial–lateral and dorsal–ventral grid, based on imaging of acute slices immediately after recording. NAc shell and core were identified as delineated regions within the Str complex. The CPu was divided into anterior and posterior subregions at AP (anterior–posterior) -0.5 mm from bregma; anterior CPu was AP ≥ −0.5 mm and posterior CPu AP < −0.5 mm, based on clusters of dopamine neuron synaptic input to ChIs (Chuhma et al., 2023). AP −0.5 mm is at approximately the center of the globus pallidus. The anterior CPu was further divided into medial and lateral CPu at ML (medial–lateral) 2 mm from the midline as an approximated division using a dorso–ventral straight line based on functional subdivisions (Berendse et al., 1992; Pennartz et al., 2009; Liljeholm and O’Doherty, 2012). Subregion delineations are illustrated in Figure 1A.

### 2.3 Data analysis

All data were analyzed with Axograph X (Axograph; RRID:SCR_014284). Parameter measurement methods are shown in Supplementary Figure S1. Resting membrane potential (Vrest) was determined as the average potential of a 1.2-s window without current injection. For spontaneously active cells (ChIs), action potentials (APs) were truncated and average potential was measured. Input impedance (input R) was measured as peak voltage amplitude, which was divided by injected current with −50 pA current injection. Membrane time constant (memb tau) was measured with −50 pA current injection by single exponential fitting from the trace onset. Sag ratio was calculated as the ratio of plateau amplitude to peak amplitude at −400 pA current injection; a ratio of 1 indicates no voltage sag. For spontaneous firing (s-firing) and input–output curve, APs were automatically detected with threshold crossing at 0 mV. AP threshold was determined as the point change in potential exceeding 10 mV/ms. AP height was an average of amplitude from AP threshold to peak for each AP, and width was an average of width at 50% of AP amplitudes. After-hyperpolarization (AHP) amplitude was an average of amplitude from AP threshold to AHP peak for each AHP, and AHP width was an average of width at 50% of AHP amplitudes. Rheobase was the minimum amount of current injected to evoke firing; it was 0 mV for spontaneously active cells. Supplementary Figure S1 shows the schema for parameter measurement. Firing frequencies were plotted against injected current amplitude (input–output curve), and the slope of a linear fit of the curve was obtained as an indicator of how firing increased with injected current. Only the injection range of 0–300 pA of input–output curves is shown in a figure; all data are available in data repository. Since SPNs showed firing after ramp of voltage at rheobase, we measured delay from the onset of step current injection to the peak of the first AP as discriminator of SPNs.

To visualize the distribution of membrane properties for each cell, with 14 parameters in two-dimensional space, dimension reduction was performed with principal component analysis (PCA). PCA was performed with *prcomp* function in the *stats* package of R4.3.1 (RRID: SCR_001905), with variable standardization (center = True, scale. = True, in *prcomp*). Eigenvalues, percentages explaining the original data variables, and cumulative percentages for principal components (PCs) for each cell type are shown in Supplementary Table S2. Quality of representation of each variable in PC1-5 and contribution of variables to PC1-5 in each cell type are shown in Supplementary Figure S2. Plots were carried out for the first and second PCs (PC1 and PC2) with *fviz_pca_biplot* function in the *factoextra* 1.0.7 package (RRID:SCR_016692). To examine differences between dSPNs and iSPNs, Euclidean distance of membrane parameters between all possible dSPN-iSPN pairs was calculated for each Str subregion. To avoid non-uniform contributions, parameters were normalized to minimum and maximum values. With k membrane parameters per cell and m dSPNs and n iSPNs in a subregion, Euclidean distance (D) of data between the i th dSPN (1 ≤ i ≤ m) and j th iSPN (1 ≤ j ≤ n) is given as follows:


$$D_{ij}=\sqrt{\overset{k}{\underset{g=1}{\sum }}{\left(dSPN_{i}\left(g\right)-iSPN_{j}\left(g\right)\right)}^{2}}$$

Where g means the gth measured parameter (1 ≤ g ≤ k).

Therefore, mean Euclidean distance in this subregion is as follows:


$$\mathrm{Mean}\;D=\frac{\sum_{i=1}^{m}\sum_{j=1}^{n}D_{ij}}{mn}$$

Computation of the Euclidean distance was performed with *rdist* function in the *fields* 15.2 package in R4.3.2.

### 2.4 Statistical analysis

All statistical analyses were conducted in R4.3.1 or R4.3.2. ANOVAs were performed using packages *afex* 1.3–0 (RRID:SCR_022857; for Type III SS ANOVA) and *emmeans* 1.8.7 (RRID:SCR_018734; for *post hoc* comparison). Two-way ANOVA was used for location or cell type heterogeneity of membrane property parameters, and three-way mixed model ANOVA was used for input–output curve. When sphericity was violated, the Greenhouse–Geisser correction was applied. For *post-hoc* multiple pairwise comparison, Tukey’s exact method was used for adjustment.

To examine regional heterogeneity with simultaneous consideration of multiple parameters, we performed one-way multivariate analysis of variance (MANOVA). Since parameters were in different units and MANOVA requires the same variances for all variables (homoscedasticity), measured values of each parameter were standardized to a distribution with mean = 0 and SD = 1 (z score). MANOVA was performed with *manova* function in the *stats* package. We used Pillai’s Trace as MANOVA statistic because Pillai’s Trace is more robust to the violation of MANOVA assumption (e.g., multivariate normality, homoscedasticity) and unequal sample sizes. A higher value of Pillai’s Trace indicates greater differences among groups. Effect size (partial eta squared) was calculated with *eta_sqared* function in the *effectsize* 0.8.6 package. Since MANOVA compares a composite dependent variable calculated from the array of dependent variables, it uses approximated F with model degrees of freedom. Contribution of each single parameter to location difference in MANOVA was examined by one-way ANOVA using *summary.aov* function in the *stats* package. For multivariate *post-hoc* analysis to identify significantly different locations, linear discriminant analysis (LDA) was performed by *lda* function in *MASS* 7.3–60 (RRID:SCR_019125) using only parameters with significant location effects. To evaluate location difference for LDA results, weighted Euclidean distance of group (location) mean of linear discriminants (LDs) between all possible location pairs for each cell type was calculated. The proportion of traces (contribution to discriminability) of LDs was used as weight. Since there are 5 locations to compare, the number of LDs is 4 (number of classes minus 1) for each cell type, and all 4 LDs were used for weighted Euclidean distance calculation. Since LDs were calculated separately by cell type, each cell type had an unique combination of LDs and proportion of traces. When a cell type has four LDs with the proportion of traces (PT) of PT_1_ to PT_4_, weighted Euclidean distance between location/subregion means of subregion A with LD means of a_1_ to a_4_ and subregion B with LD means of b_1_ to b_4_ is given as follows:


$$\mathrm{Location}\;\mathrm{mean}\;D=\sqrt{\overset{4}{\underset{LD=1}{\sum }}{\left(\left(a_{LD}-b_{LD}\right)PT_{LD}\right)}^{2}}$$

All tests were two-tailed, and the significance was set at *p* < 0.05. Data are presented as mean ± SEM unless otherwise noted.

## 3 Results

The intrinsic membrane properties of dSPNs, iSPNs, ChIs, and FSIs were recorded under whole cell current clamp throughout the Str (NAc and CPu; Figure 1). The Str was divided into five subregions, namely, NAc shell, NAc core, medial (med) CPu, lateral (lat) CPu, and posterior (post) CPu (Figure 1A). dSPNs, iSPNs, and FSIs were identified by the expression of genetic markers prior to recording, while ChIs were visualized based on their larger soma size, and their identify was confirmed by shallow resting membrane potential, tonic firing, and voltage sag with negative current injection; all mouse Str neurons with these characteristics are immunoreactive for choline acetyltransferase (ChAT) and are identified as ChIs (Chuhma et al., 2014).

**Figure 2:**
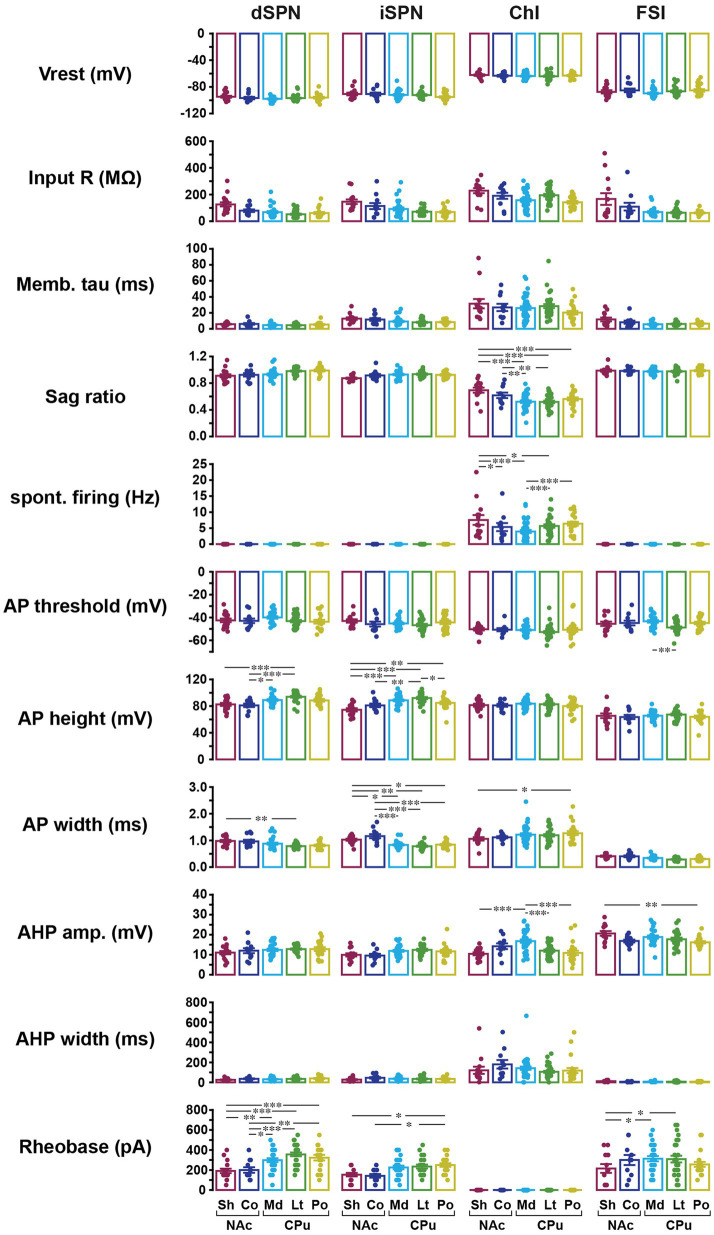
Basic membrane properties of four striatal cell types in five subregions. Basic membrane properties were measured for dSPN, iSPN, ChIs, and FSIs in the NAc shell (Sh; dark red), NAc core (Co; dark blue), medial (Md) CPu (cyan), lateral (Lt) CPu (green), and posterior (Po) CPu (dark yellow). The 11 membrane properties measured were resting membrane potential (Vrest), input resistance (Input R), membrane time constant (Memb tau), sag ratio, spontaneous firing, action potential (AP) threshold, AP height, AP width, after-hyperpolarization (AHP) amplitude, AHP width, and rheobase. Dots indicate single cells; bars mean ± S.E.M. *, **, *** indicate *p* < 0.05, *p* < 0.01, and *p* < 0.001, respectively, in two-way ANOVA (location and cell-type interaction). See Supplementary Figure S1 for measurement methods. See Table 1 for numbers of cells recorded by cell-type and subregion, Table 2 for ANOVA results, and Supplementary Table S3 for *post-hoc* analysis of interactions and location effects.

A step current injection (duration of 700 ms, 50 pA step) was delivered, and the following parameters measured: resting membrane potential (Vrest), input impedance (input R), membrane time constant (memb tau), voltage sag ratio with −400 pA current injection calculated as plateau divided by peak voltage (smaller ratio indicates more sag), spontaneous firing, action potential threshold (APTH), AP height, AP width at 50% of peak, after-hyperpolarization amplitude (AHP amp), AHP width at 50% of peak, and rheobase (minimum current required to fire the neuron; Figure 2; Supplementary Figure S1). At least 100 cells of each cell type, in total 432 cells, were recorded, (Table 1). Since ChIs, as well as some GABAergic interneurons, are spontaneously active in slice (Goldberg and Wilson, 2016; Tepper et al., 2018), and spontaneous glutamate and GABA release shows regional variation (Ma et al., 2012), glutamate, GABA, and acetylcholine (ACh) receptors were continuously blocked.

**Table 1:** Numbers of cells recorded.

	NAc shell	NAc core	med CPu	lat CPu	post CPu	Total
dSPN	17	12	30	31	19	109
iSPN	14	12	31	23	21	101
ChI	14	11	40	33	23	121
FSI	13	11	27	28	22	101

All basic membrane parameters (Figure 2) showed significant cell type differences (two-way ANOVA, cell type effect, all *p* < 0.001; Table 2). Significant location and cell-type interactions were observed in most parameters, except Vrest, input R, memb tau, and AHP width (Table 2). Among these parameters without significant interactions, input R and memb tau showed significant location effects (Table 2) and regional heterogeneity independent of cell type. *Post-hoc* comparisons showed that these significant interactions and location effects were mainly due to differences between the NAc and CPu, particularly between the NAc shell and CPu subregions (Figure 2; Supplementary Table S3). Input R and memb tau showed a significant difference between NAc shell and CPu; particularly, NAc shell neurons showed a higher input R compared with all CPu subregions. SPNs showed robust regional heterogeneity in AP height (smaller in the NAc), AP width (larger in NAc), and rheobase (smaller in NAc). ChIs showed robust differences in sag ratio (ratio was larger in the NAc, reflecting less sag), spontaneous firing (higher in NAc shell and lower in med CPu), and AHP amp (larger in med CPu), while regional heterogeneity was less for FSIs. Since only ChIs were spontaneously active, spontaneous firing frequency was zero in the other three types of neurons, and rheobase in ChIs was invariably zero, so there was no regional heterogeneity in these parameters.

**Table 2:** ANOVA results for main parameters.

	Effect	*df*	MSE	*F*	ges	*p*-value
Vrest	Cell_type	3, 412	34.35	608.23	0.816	<0.001 ***
	Location	4, 412	34.35	1.60	0.015	0.174
	Cell_type:location	12, 412	34.35	1.19	0.033	0.290
Input R	Cell_type	3, 412	3280.23	64.48	0.320	<0.001 ***
	Location	4, 412	3280.23	22.24	0.178	<0.001 ***
	Cell_type:location	12, 412	3280.23	1.50	0.042	0.119
Memb. tau	Cell_type	3, 412	71.98	122.41	0.471	<0.001 ***
	Location	4, 412	71.98	3.75	0.035	0.005 **
	Cell_type:location	12, 412	71.98	1.15	0.032	0.318
Sag ratio	Cell_type	3, 412	0.01	502.61	0.785	<0.001 ***
	Location	4, 412	0.01	1.93	0.018	0.104
	Cell_type:location	12, 412	0.01	6.47	0.159	<0.001 ***
Spont. firing	Cell_type	3, 412	3.44	229.42	0.626	<0.001 ***
	Location	4, 412	3.44	2.8	0.026	0.026 *
	Cell_type:location	12, 412	3.44	3.03	0.081	<0.001 ***
AP threshold	Cell_type	3, 412	33.42	37.74	0.216	<0.001 ***
	Location	4, 412	33.42	4.24	0.040	0.002 **
	Cell_type:location	12, 412	33.42	0.91	0.026	0.534
AP height	Cell_type	3, 412	67.99	126.97	0.480	<0.001 ***
	Location	4, 412	67.99	13.13	0.113	<0.001 ***
	Cell_type:location	12, 412	67.99	2.78	0.075	0.001 **
AP width	Cell_type	3, 412	0.04	284.76	0.675	<0.001 ***
	Location	4, 412	0.04	6.01	0.055	<0.001 ***
	Cell_type:location	12, 412	0.04	4.19	0.109	<0.001 ***
AHP amp	Cell_type	3, 412	13.50	62.36	0.312	<0.001 ***
	Location	4, 412	13.50	5.61	0.052	<0.001 ***
	Cell_type:location	12, 412	13.50	4.65	0.119	<0.001 ***
AHP width	Cell_type	3, 412	3251.34	88.61	0.392	<0.001 ***
	Location	4, 412	3251.34	1.41	0.014	0.230
	Cell_type:location	12, 412	3251.34	1.10	0.031	0.360
Rheobase	Cell_type	3, 412	9754.35	164.99	0.546	<0.001 ***
	Location	4, 412	9754.35	9.33	0.083	<0.001 ***
	Cell_type:location	12, 412	9754.35	2.69	0.073	0.002 **

The results are given of two-way ANOVA (location and cell-type) for each membrane parameter shown in Figure 2. Cell-type effect, location effect, and location and cell-type interaction are shown. *df*, degree of freedom; MSE, mean squared error; ges, general eta squared (effect size). *, **, *** Indicate *p* < 0.05, *p* < 0.01, and *p* < 0.001, respectively. See Supplementary Table S3 for *post-hoc* test results.

**Figure 3:**
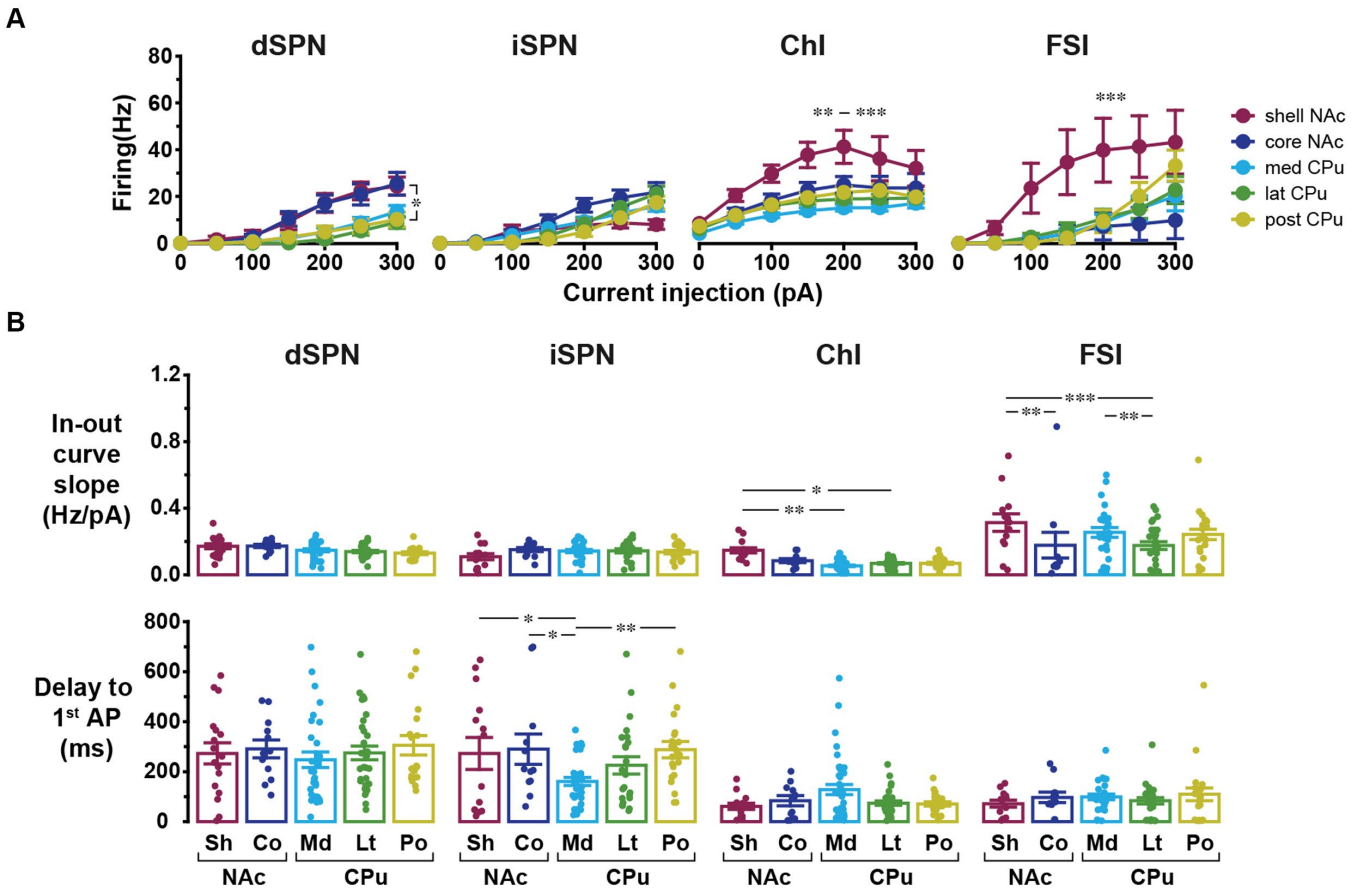
Input–output curves and related parameters of four cell types in five locations. **(A)** Input–output curves for dSPNs, iSPNs, ChIs, and FSIs in NAc shell (dark red), NAc core (dark blue), medial CPu (cyan), lateral CPu (green), and posterior CPu (dark yellow). Each point indicates mean ± S.E.M. *In dSPN panel indicates *p* < 0.05 comparison between NAc shell and lateral CPu in three-way mixed ANOVA. **, ***In ChI panel indicated that NAc shell was significantly different from posterior CPu with *p* < 0.01 and from medial and lateral CPus with *p* < 0.001. ***In FSI panel indicated that NAc shell was significantly different from all other subregions with *p* < 0.001. **(B)** Slope of linear part of input–output curve (top) and delay to the first action potential (AP) from the onset of current injection at rheobase (bottom). Dots indicate individual cells and bars indicate means ± S.E.M. *, **, and *** Indicate *p* < 0.05, *p* < 0.01, and *p* < 0.001, respectively, in two-way ANOVA (location and cell-type interaction). See Table 3 and Supplementary Table S4 for results of ANOVA and *post-hoc* analysis, respectively.

In addition to basic membrane properties, the relationship between depolarizing current injection and evoked firing, the input–output curve, was examined (Figure 3). For ChIs and FSIs, the input–output curve in NAc shell showed a steeper rise and required less current injection to reach saturation as compared with other subregions (three-way mixed ANOVA; Figure 3A; Table 3; Supplementary Table S4). For iSPNs, the input–output curve in the NAc shell appeared to reach saturation (Figure 3A) presumably due to depolarization block. To quantitate the steepness of the rise of the curves, we fit the linear part of the input–output curve and compared the slopes of the fitted lines (Supplementary Figure S1; Figure 3B). The slope differed significantly among cell types (two-way ANOVA, *p* < 0.001) and also showed significant cell type and location interactions (*p* = 0.001); most significant differences were between NAc shell and other regions (Supplementary Table S4). SPNs responded to positive current injection with a voltage ramp and delayed firing (Kreitzer, 2009). We also measured the delay from the start of step current injection to the first AP (at the rheobase) as another possibly regionally heterogeneous parameter particularly for SPNs (Supplementary Figure S1; Figure 3B). The delay was significantly different among cell types (two-way ANOVA, *p* < 0.001). Significant location and cell type interactions were observed (*p* = 0.025), with the significant difference between med CPu and other locations for iSPNs (Table 3; Supplementary Table S4).

**Table 3:** ANOVA results for input–output curves and related parameters.

Input–output curve					
Effect	*df*	MSE	*F*	ges	*p*-value
Cell_type	3, 412	715.42	29.93	0.108	<0.001 ***
Location	4, 412	715.42	12.34	0.062	<0.001 ***
Cell_type: location	12, 412	715.42	3.38	0.052	<0.001 ***
Injection	1.80, 741.04	318.53	189.85	0.170	<0.001 ***
Cell_type: injection	5.40, 741.04	318.53	8.04	0.025	<0.001 ***
Location: injection	7.19, 741.04	318.53	4.82	0.020	<0.001 ***
Cell_type: location: injection	21.58, 741.04	318.53	2.36	0.030	<0.001 ***

Related parameters						
	Effect	*df*	MSE	*F*	ges	*p*-value
Slope	Cell_type	3, 412	0.01	44.19	0.243	<0.001 ***
	Location	4, 412	0.01	3.54	0.033	0.007 **
	Cell_type: location	12, 412	0.01	2.74	0.074	0.001 **
Delay	Cell_type	3, 412	15615.43	63.15	0.315	<0.001 ***
	Location	4, 412	15615.43	1.06	0.010	0.376
	Cell_type: location	12, 412	15615.43	1.98	0.054	0.025 *

The results are given for three-way mixed ANOVA of input–output curve (location, cell-type, and current injection amount), and two-way ANOVA of input–output curve slope and delay to the first action potential at rheobase (location and cell-type) shown in Figure 3. *df*, degree of freedom; MSE, mean squared error; ges, general eta squared (effect size). *, **, *** Indicate *p* < 0.05, *p* < 0.01, and *p* < 0.001, respectively. See Supplementary Table S4 for *post-hoc* tests.

**Figure 4:**
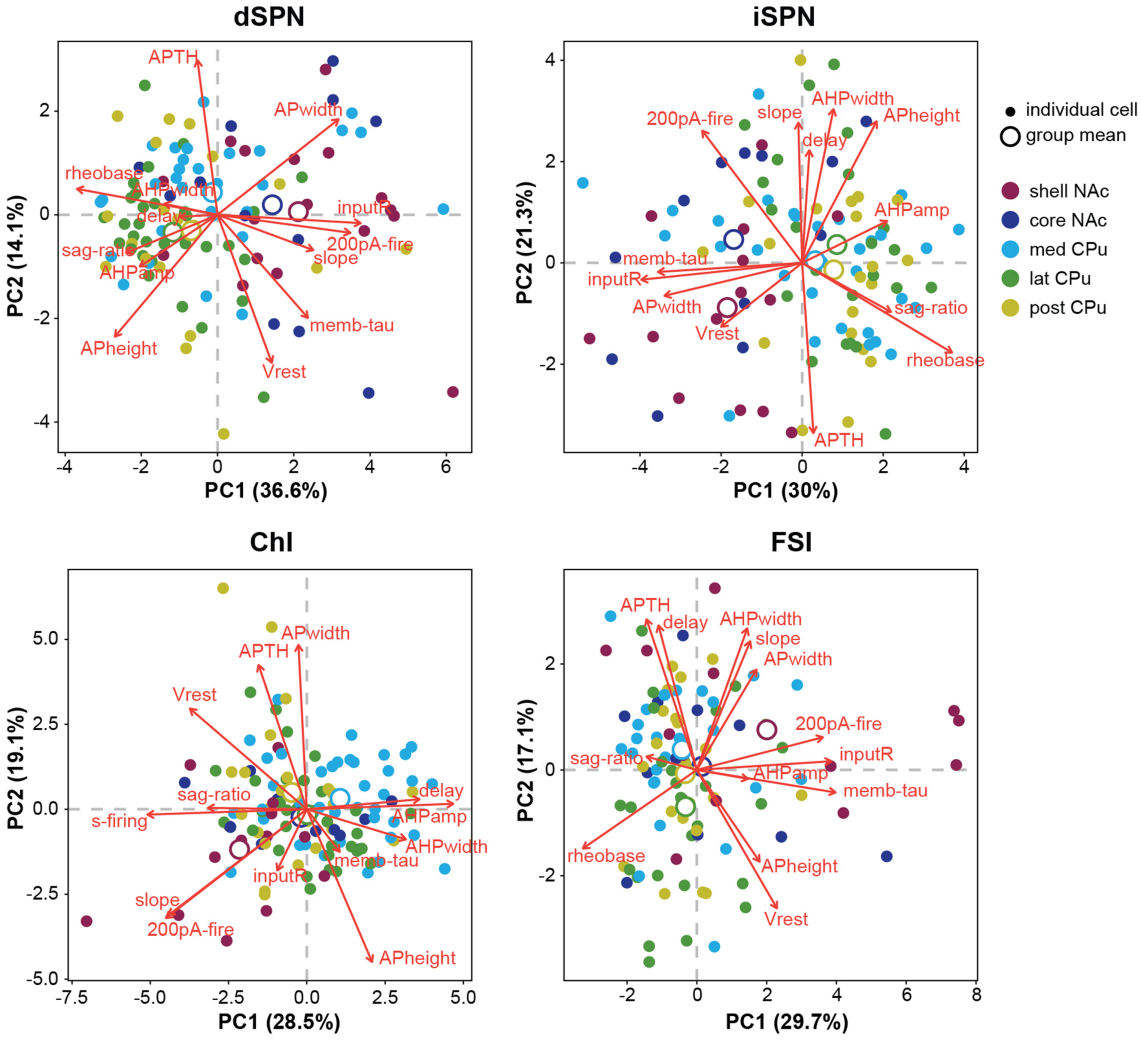
Principal component analysis of subregional distribution of membrane parameters by cell-type. Principal component analysis (PCA) is shown by cell type, with colors indicating regional localization of cells. Smaller closed circles indicate individual cells; larger open circles indicate subregion (group) means. Percentage of variance explained by each PC is in the axis label. Red arrows indicate direction and amount of the contribution (length of arrows) of parameters (red letters) to PC1 and PC2. Projecting arrows to the respective axes show the contribution to the PC.

To summarize visually the observations in multiple parameters in two dimensions, we performed a principal component analysis (PCA) for dimension reduction (Figure 4). To include observations of the input–output curve, we used firing frequency elicited with 200-pA current injection (200-pA firing) to avoid reaching saturation. PCA cannot be performed on a variable with a single value; therefore s-firing of SPNs and FSIs and rheobase of ChIs were dropped (the values were 0), making the total number of parameters 13. PCA is a data exploratory method to improve data visualization by reducing the number of variables while minimizing information loss (Jolliffe and Cadima, 2016). To do so, new sets of variables are found, which are linear combinations of the original variables, maximizing retained original variance (principal components; PCs). The PC with most of the original variance becomes the first PC (PC1) and the next largest becomes PC2. Plots of PC1 and PC2 for each cell, by cell type, and means for each location (group mean) are shown in Figure 4. PC1 and PC2 retained approximately 50% of the variance. Red arrows in Figure 4 indicate the contribution of the parameters to PC1 and PC2 and their direction; longer arrows aligned with either axis indicate a greater contribution to that PC, with direction indicating positive or negative correlation (Supplementary Table S2; Supplementary Figure S2). Judging from the position of location means and the results of single parameter ANOVAs (Figures 2, 3), both SPN types in the NAc shell and core were different from the CPu, based on higher input R and smaller rheobase, higher 200-pA firing in dSPNs, and greater AP width in iSPNs. The slope of the input–output curve was steeper in the NAc in dSPNs, but contributed more to PC2 in iSPNs, and differed between NAc shell and core subregions. For ChIs and FSIs, the NAc shell differed from other subregions, which was characterized by higher input R, greater 200-pA firing, and input–output curve slope. Compared with other cell types, ChIs in the medial CPu showed a more skewed distribution, characterized by lower s-firing, lower sag ratio, and greater AHP amp (Figure 4).

We assessed regional heterogeneity (location effect) considering all measured parameters by multivariate analysis of variance (MANOVA) for each cell type (Table 4). For all cell types, location effect (regional heterogeneity) was very strong (Pillai’s Trace 0.975–1.164, partial Eta squared 0.24–0.29, *p* < 0.001). *Post-hoc* tests for contribution of each parameter to regional heterogeneity (Table 4) confirmed the results of the single parameter analyses (Tables 2, 3), with strong location effects for most variables, except for Vrest, APTH, AHP width, and delay to the first AP.

**Table 4:** MANOVA for location effects.

Main MANOVA								
Cell type	Location *df*	Residual *df*	Pillai	Approx. *F*	Num *df*	Den *df*	*p*-value	Partial eta^2^
dSPN	4	104	0.983	2.38	52	380	< 0.001 ***	0.25
iSPN	4	96	1.164	2.75	52	348	< 0.001 ***	0.29
ChI	4	116	0.975	2.65	52	428	< 0.001***	0.24
FSI	4	96	1.016	2.28	52	348	< 0.001 ***	0.25

Single variable effects on difference between locations								
	dSPN		iSPN		ChI		FSI	
	*F*	*p*	*F*	*p*	*F*	*p*	*F*	*p*
Vrest	1.23	0.30	1.59	0.18	0.52	0.72	1.24	0.30
Input R	9.94	< 0.001 ***	5.98	< 0.001 ***	5.86	< 0.001 ***	6.60	< 0.001 ***
Memb tau	1.94	0.11	3.39	0.012 *	1.55	0.19	5.73	< 0.001 ***
Sag ratio	5.21	< 0.001 ***	3.40	0.012 *	7.16	< 0.001 ***	0.31	0.87
APTH	1.46	0.22	1.05	0.38	0.80	0.53	3.41	0.012 *
AP height	8.43	< 0.001 ***	11.5	< 0.001 ***	0.92	0.46	0.63	0.64
AP width	5.68	< 0.001 ***	17.4	< 0.001 ***	1.35	0.26	9.51	< 0.001 ***
AHP amp	1.00	0.41	3.03	0.021 *	10.0	< 0.001 ***	3.57	0.0093 **
AHP width	2.79	0.030 *	1.39	0.24	1.33	0.27	1.99	0.10
s-firing	N/A	N/A	N/A	N/A	3.57	0.0088 **	N/A	N/A
Rheobase	9.71	< 0.001 ***	5.19	< 0.001 ***	N/A	N/A	1.33	0.26
200pA fire	8.93	< 0.001 ***	2.48	0.049 *	11.4	< 0.001 ***	4.00	0.0048 **
Slope	3.02	0.021	1.33	0.26	20.6	< 0.001 ***	2.10	0.086
Delay	0.42	0.80	2.75	0.033 *	3.08	0.019 *	0.49	0.74

**Figure 5:**
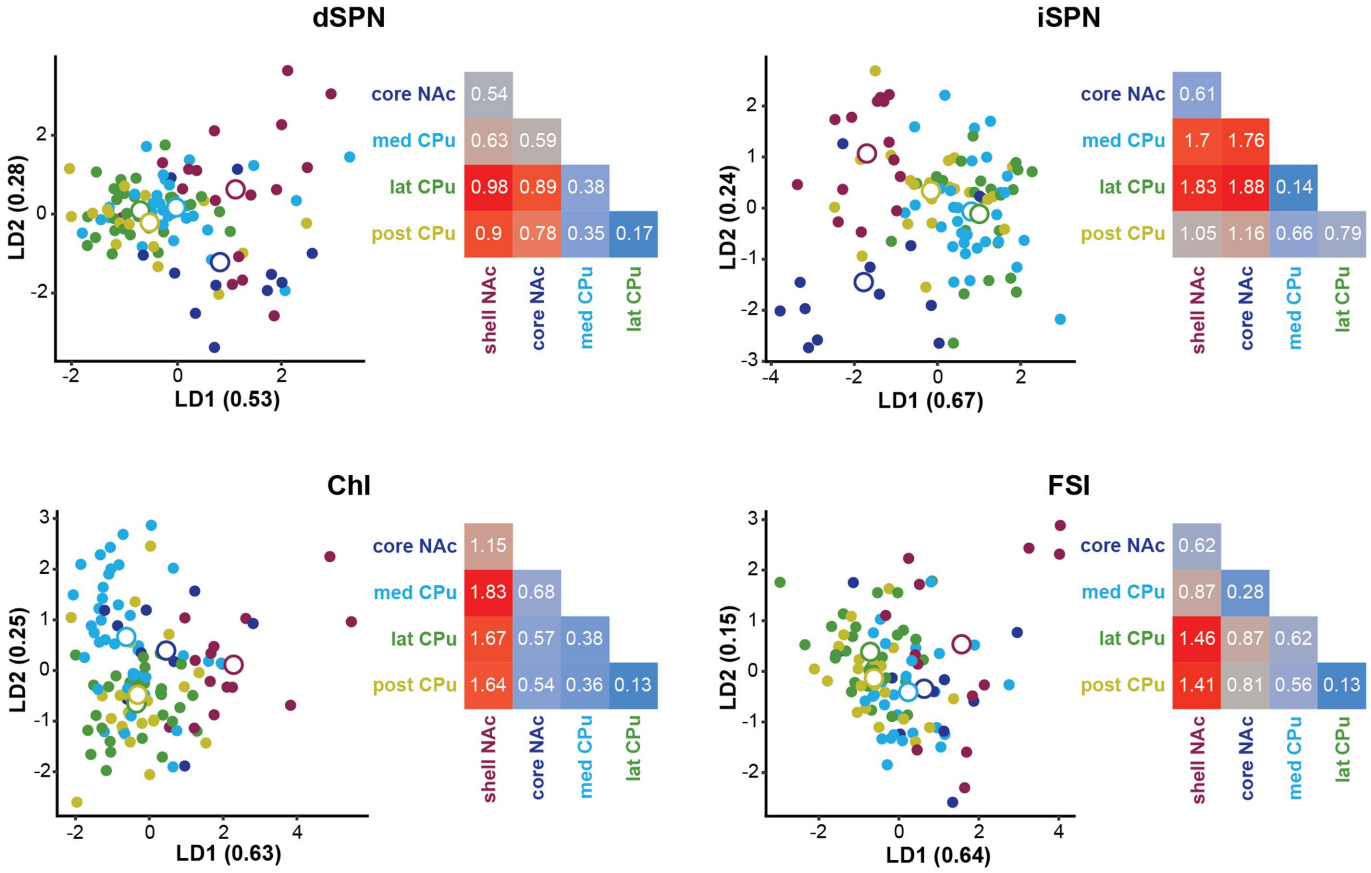
Linear discriminant analysis as *post-hoc* analysis of MANOVA location effects. Linear discriminant analysis (LDA) shows discriminability of locations in the four Str cell types using parameters with significant contribution to location differences. The first two linear discriminants (LD) were plotted. The proportion of traces separated by each LD is in the axis labels. Smaller closed circles indicate individual cells, and larger open circles indicate subregion (group) mean. Heatmaps to the right of each LDA plot give distances between two location means weighted by the proportion of traces of each LD using all four LDs for each cell type. Distances are indicated by a color gradient from maximum (red), to midpoint (gray), to minimum distance (blue), with the distance in white text. Proportions of traces and group means for all four LDs are shown in Table 5.

The results of one-way MANOVA (location effect) for each cell type. Pillai, Pillai’s Trace (MANOVA Statistic); Approx F, approximated *F* value; Num df, number of degrees of freedom in the model; Den *df*, degrees of freedom associated with the model errors; Partial eta^2^, partial eta squared (effect size). Contribution of each variable to location effects is shown below. Parameters with significant contributions to location effects were used for linear discriminant analysis shown in Figure 5. *, **, *** Indicate *p* < 0.05, *p* < 0.01, and *p* < 0.001, respectively.

To identify the subregions responsible for strong location effects, we performed a linear discriminant analysis (LDA) as a multivariate *post-hoc* analysis of MANOVA (Finch, 2009; Warne 2014; Carlson, 2017; Figure 5). LDA also achieves dimension reduction but differs from PCA; LDA finds a linear combination of features that separate two or more groups (Fisher, 1936; Hastie et al., 2009), instead of finding a linear combination of features that retain the original data information with PCA. By using these features (linear discriminant; LD), the separation among groups is maximized and the group that deviates more from the others is more easily visualized. To maximize the separation of groups for LDA, we used only parameters with significant location effects in the MANOVA (Table 4). The LD providing the greatest separation becomes the first LD (LD1), followed by the second best LD (LD2). The number of LDs is the number of groups minus 1 (i.e., 4 LDs for 5 subregions). Plots of LD1 and LD2 for individual cells and group means for each cell type are shown in Figure 5. The percentage of discriminability achieved by each LD (proportion of trace) is shown in parentheses for each axis, e.g., LD1 (0.53) indicates 53% of the discrimination (Figure 5; Table 5). To quantitate subregional differences for each cell type, we calculated Euclidean distances between group means using all four LDs weighted by the proportion of traces. The group mean Euclidean distances are shown in Figure 5 (inset heatmaps). Since LDs were calculated separately for each cell type, absolute distances between different cell types are not comparable. A larger distance indicates that the two subregions differ more, while a shorter distance indicates that the two subregions are more similar.

**Table 5:** Proportions of trace and location means of linear discriminants.

SPNs								
	dSPN				iSPN			
	LD1	LD2	LD3	LD4	LD1	LD2	LD3	LD4
Prop. trace	0.527	0.277	0.178	0.019	0.667	0.240	0.085	0.009
NAc shell	1.156	0.630	−0.341	0.048	−1.698	1.070	−0.363	−0.078
NAc core	0.858	−1.216	0.097	0.075	−1.775	−1.449	−0.013	−0.009
CPu med	0.014	0.169	0.524	−0.111	0.814	−0.067	−0.362	0.120
CPu lat	−0.675	0.093	0.005	0.156	1.007	−0.114	0.106	−0.203
CPu post	−0.497	−0.213	−0.592	−0.170	−0.158	0.338	0.668	0.103

Interneurons								
	ChI				FSI			
	LD1	LD2	LD3	LD4	LD1	LD2	LD3	LD4
Prop. trace	0.629	0.252	0.109	0.011	0.640	0.149	0.121	0.090
NAc shell	2.337	0.114	0.044	−0.101	1.569	0.535	0.074	0.222
NAc core	0.512	0.394	−0.049	0.354	0.633	−0.344	0.478	−0.629
CPu med	−0.569	0.663	0.053	−0.055	0.237	−0.409	−0.395	0.080
CPu lat	−0.294	−0.649	0.414	0.005	−0.716	0.386	−0.170	−0.192
CPu post	−0.257	−0.480	−0.689	−0.019	−0.623	−0.134	0.419	0.329

Means of linear discriminants (LDs) for each location for the four cell types are shown with proportions of traces (contribution of LD to discrimination). Number of LDs is the number of locations minus 1 (4 LDs). Plots of LD1 and LD2 are shown in Figure 5.

For dSPNs and iSPNs, NAc subregions showed recognizably longer distances compared to CPu subregions, indicating that both NAc shell and core differ from CPu subregions (Figure 5; Table 5). The CPu subregions were similar to each other for both dSPNs and iSPNs. NAc shell and core were similar to each other for iSPNs; the similarity was comparable to the CPu subregions, while NAc shell and core for dSPNs were recognizably different. Although iSPN NAc shell and core appeared to be separated by LD2, most of the discriminability was determined by LD1 (67%), and these two subregions did not differ when the weight of the contribution to discriminability was considered. For ChIs, the NAc shell was the most differentiated subregion based on distances between location mean pairs, while the other four subregions were more similar to each other. For FSIs, the NAc shell was differentiated from the CPu, particularly from lateral and posterior CPu, followed by a trend of increasing difference in cell properties with physical distance between subregions.

Taken together, all four cell types showed regional heterogeneity in membrane properties. The NAc shell differed from CPu subregions for all cell types, characterized by higher input R, lower rheobase, greater 200-pA firing, and steeper input–output curve slope. All CPu subregions showed higher similarity for all four cell types. Similarity of the NAc core to other subregions differed among cell types. For SPNs and FSIs, NAc shell and core were more similar to each other than to the CPu, while ChIs showed differences between NAc shell and core; the NAc core was more similar to the CPu subregions in ChIs. For both SPN types, the NAc core differed substantially from the CPu, while FSIs in the NAc core did not. For FSIs, the difference appeared to increase gradually with the physical distance of the subregions, while the dichotomy between NAc subregions and CPu was not as clear compared with the other cell types. Although ChIs in the med CPu showed slower spontaneous firing, accompanied by a larger and slower AHP, with more prominent sag (lower sag ratio) in individual parameter analysis, the med CPu was not recognizably different in the multivariate analysis.

**Figure 6:**
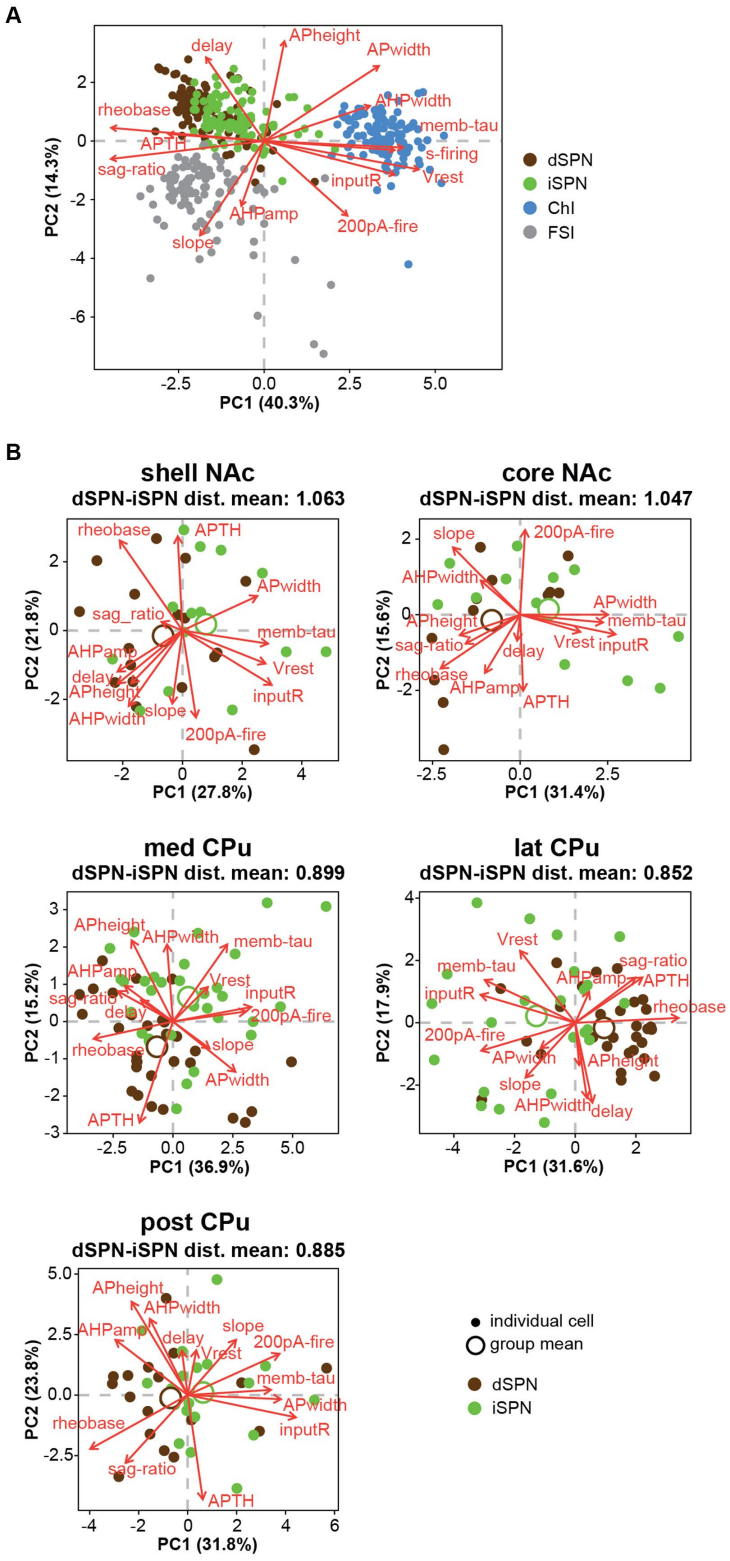
Differences between dSPNs and iSPNs in Str subregions. **(A)** Principal component analysis (PCA) of all recorded Str neuron types is shown in plots of the first two PCs. Single cells are shown as filled circles, with cell types shown by different colors. Red arrows indicate the direction and contribution of parameters to PC1 and PC2. ChIs, FSIs, and SPNs are clearly separable, while the two subtypes of SPNs largely overlap. **(B)** PCA of dSPNs (brown) and iSPNs (green) in the five Str subregions. Large open circles indicate group (each subregion each cell type) means. Mean Euclidean distances (dist) of normalized parameters among all dSPN-iSPN pairs in each Str subregion are shown above each PCA plot, indicating differences between dSPNs and iSPNs.

PCA of all recorded Str neurons revealed that ChIs and FSIs were substantially different from SPNs, and these three neuron types were less likely to have similar properties (Figure 6A). However, dSPN and iSPN distributions were recognizabe but not totally overlapping (Figure 6A), and similarity between dSPNs and iSPNs also showed regional heterogeneity. Visual summaries of parameter distribution for dSPNs and iSPNs by subregion using PCA are shown in Figure 6B. To detect smaller differences, two-way ANOVA for each parameter applied only to dSPN and iSPN data, revealed significant differences between dSPNs and iSPNs (cell-type effect), and this was observed for most parameters, except for AHP width, 200-pA firing, and delay to the first action potential, suggesting that most of the parameters differed between dSPNs and iSPNs across the Str subregions (Table 6). Judging from single parameter graphs (Figure 2), dSPNs showed deeper Vrest, lower input R, shorter memb tau, and higher rheobase, suggesting that dSPNs were generally harder to excite than iSPNs. Location and cell type interactions were observed for AP width, 200-pA firing, and input–output curve slope (Table 6) due to a significant difference between AP width in the NAc core, 200-pA firing in the NAc shell and lateral CPu, and input–output curve slope in the NAc shell (Table 7). The significant interaction indicates that the regional heterogeneity of differences between dSPNs and iSPNs was greatest in the NAc shell. Since 200-pA firing did not show a cell-type effect but did show a significant cell-type x location interaction, firing response to injected current differed between subtypes only in the NAc shell and lateral CPu. To examine whether the 200-pA point was a good measure of the input–output curve, we performed a statistical analysis of dSPN and iSPN input–output curves (Supplementary Table S5). Three-way mixed ANOVA revealed a significant location and cell-type interaction (*p* = 0.005), and no significant cell-type effect (*p* = 0.67). *Post-hoc* comparison revealed a cell-type effect (dSPN-iSPN difference) only in the NAc shell (*p* = 0.005) and lateral CPu (*p* = 0.004); significant differences were observed in the same subregions as 200-pA firing, suggesting that the 200-pA firing point reflects regional heterogeneity of SPN input–output curves.

**Table 6:** ANOVA results for SPN subtype differences.

	Effect	*df*	MSE	*F*	ges	*p*-value
Vrest	Cell type	1, 200	31.45	27.10	0.119	<0.001 ***
	Location	4, 200	31.45	1.43	0.028	0.225
	Location:cell_type	4, 200	31.45	1.43	0.028	0.228
Input R	Cell type	1, 200	2215.81	9.39	0.045	0.002 **
	Location	4, 200	2215.81	14.49	0.225	<0.001 ***
	Location:cell_type	4, 200	2215.81	0.35	0.007	0.847
Memb. tau	Cell type	1, 200	11.91	87.21	0.304	<0.001 ***
	Location	4, 200	11.91	4.98	0.091	<0.001 ***
	Location:cell_type	4, 200	11.91	1.79	0.133	0.133
Sag ratio	Cell type	1, 200	0.00	11.87	0.0.56	<0.001 ***
	Location	4, 200	0.00	6.88	0.121	<0.001 ***
	Location:cell_type	4, 200	0.00	2.03	0.0.39	0.092
AP threshold	Cell type	1, 200	35.29	8.48	0.0.41	0.004 **
	Location	4, 200	35.29	1.36	0.0.27	0.249
	Location:cell_type	4, 200	35.29	1.28	0.025	0.280
AP height	Cell type	1, 200	67.92	5.62	0.0.27	0.019 *
	Location	4, 200	67.92	19.16	0.277	<0.001 ***
	Location:cell_type	4, 200	67.92	1.34	0.026	0.256
AP width	Cell type	1, 200	0.02	3.92	0.019	0.049 *
	Location	4, 200	0.02	19.51	0.281	<0.001 ***
	Location:cell_type	4, 200	0.02	2.74	0.052	0.030 *
AHP amp	Cell type	1, 200	9.62	6.18	0.030	0.014 *
	Location	4, 200	9.62	3.35	0.063	0.011 *
	Location:cell_type	4, 200	9.62	0.54	0.011	0.709
AHP width	Cell type	1, 200	290.42	0.94	0.005	0.333
	Location	4, 200	290.42	2.76	0.052	0.029 *
	Location:cell_type	4, 200	290.42	1.01	0.020	0.404
Rheobase	Cell type	1, 200	9260.81	26.54	0.117	<0.001 ***
	Location	4, 200	9260.81	13.71	0.215	<0.001 ***
	Location:cell_type	4, 200	9260.81	1.10	0.021	0.359
200 pA fire	Cell type	1, 200	108.45	0.01	<0.001	0.934
	Location	4, 200	108.45	7.65	0.133	<0.001 ***
	Location:cell_type	4, 200	108.45	3.39	0.063	0.010 *
Slope	Cell type	1, 200	0.00	4.67	0.023	0.032 *
	Location	4, 200	0.00	1.32	0.026	0.264
	Location:cell_type	4, 200	0.00	2.80	0.053	0.027 *
Delay	Cell type	1, 200	26096.85	1.73	0.009	0.190
	Location	4, 200	26096.85	2.57	0.049	0.039 *
	Location:cell_type	4, 200	26096.85	0.58	0.012	0.675

The results of two-way ANOVA (location and cell-type) for each parameter for only the two subtypes of SPNs. Cell-type effect, location effect, and location and cell-type interaction are shown. *df*, degree of freedom; MSE, mean squared error; ges, general eta squared (effect size). *, **, *** Indicate *p* < 0.05, *p* < 0.01, and *p* < 0.001, respectively. See Table 7 for *post-hoc* test results interactions.

**Table 7:** *Post-hoc* comparison of location and cell-type interactions for SPNs.

	NAc shell		NAc core		med CPu		lat CPu		post CPu	
	*t*-ratio	*p*	*t*-ratio	*p*	*t*-ratio	*p*	*t*-ratio	*p*	*t*-ratio	*p*
AP width	−0.85	0.40	−3.10	0.002 **	1.13	0.26	0.05	0.96	−0.58	0.56
200pA fire	2.55	0.011 *	0.20	0.84	−1.65	0.10	−2.31	0.022 *	−0.005	0.99
Slope	3.50	<0.001 ***	1.05	0.30	0.38	0.70	−0.28	0.78	−0.40	0.69

*Post-hoc* pairwise comparison between location and cell-type interactions is shown in Table 6.

When SPNs were analyzed in subregions, the number of cells became limited, so a MANOVA could not be conducted. Instead, we calculated Euclidean distances of normalized parameters for all dSPN-iSPN pairs in each subregion, considering all parameters together. Euclidean distance provides a measure of how membrane properties differ for a given dSPN-iSPN pair. The average of the distances provides a measure of the difference of dSPN and iSPN properties in the subregion. The average Euclidean distances (Figure 6B) in the NAc shell (1.063) and NAc core (1.047) were quite similar, while those in the three CPu regions differed substantially from the NAc but were similar (med CPu: 0.899, lat CPu: 0.852, and post CPu: 0.885). Thus, differences between dSPNs and iSPNs show regional heterogeneity due to differences between NAc and CPu SPNs, as dSPNs and iSPNs differed more in the NAc than in the CPu.

## 4 Discussion

Str neuron types have distinctive membrane properties that vary by subregion. All four cell types showed regional heterogeneity. The most distinctive subregion was the NAc shell, characterized by cells with higher input R and excitability; lower rheobase in SPNs and FSIs, higher spontaneous firing rate and smaller voltage sag in ChIs, and more firing with the same amount of current injection in dSPNs, ChIs, and FSIs. The three CPu subregions showed more similarity in all four cell types as compared with the NAc core. For both SPN subtypes, the NAc core was similar to the NAc shell but differed from the CPu, characterized by shorter AP and lower rheobase but not by input R. For ChIs, the NAc core was more similar to the CPu. For FSIs, more distant subregions showed more differences, and the NAc shell differed less from the medial CPu than the other cell types. We also examined regional heterogeneity of similarity between dSPNs and iSPNs; dSPNs and iSPNs differed in most parameters, except AP threshold and input–output curve slope. Among the different parameters, regional differences were observed in AP width (in the NAc core), 200-pA firing (in the NAc shell and lat CPu), and input–output curve slope (in the NAc shell), indicating that differences in the NAc contribute the most to regional heterogeneity. Indeed, differences between dSPN-iSPN pairs in each subregion revealed that dSPNs and iSPNs in the NAc differ more than in the CPu.

All four neuron types were more excitable in the NAc shell, which was reflected in greater firing to the same step current injection, and a steeper input–output curve. This is related to higher input R, which would increase in response to the same synaptic input. Similarly in rats, SPNs in the NAc shell are more excitable than those in the NAc core (Maria-Rios et al., 2023). The shallower slope of the input–output curve for iSPNs in the NAc shell could be due to depolarization block; if so, iSPNs may have a smaller dynamic range than dSPNs in the NAc shell. The NAc core was also distinguished by higher excitability without higher input R for both SPN subtypes.

Since the most posterior part of the CPu shows distinct anatomical features and functions (Menegas et al., 2015, 2017; Miyamoto et al., 2019; Valjent and Gangarossa, 2021), we expected that neurons in the posterior CPu would differ from other CPu subregions. However, membrane properties in the posterior CPu did not differ from those in other CPu subregions for any cell type, suggesting that individual cellular computation in the posterior part of the CPu is likely to be similar to other CPu subregions. Although macro cytoarchitecture appears not to affect intrinsic excitability of individual neurons, anatomical features of individual neurons, particularly differences in dendrite length and complexity, affect intrinsic excitability of Str neurons (Gertler et al., 2008; Plenz and Wickens, 2016). dSPNs have longer total dendritic length, more primary dendrites, and more branch points and tips compared with iSPNs (Gertler et al., 2008). Computer simulation of model iSPNs showed that fewer primary dendrites and fewer branch points increase excitability (Gertler et al., 2008). Although a systematic analysis of dendritic morphology across the striatum has not been performed, differences in SPN dendritic arborization among Str subregions could contribute to regional heterogeneity.

Membrane properties/excitabilities are determined mostly by the type and amount of ion channels expressed. Single cell RNA seq has revealed a differential pattern of ion channel expression in different cell types in the dorsal CPu (Muñoz-Manchado et al., 2018). Spontaneous pace making firing of ChIs is determined by Cav2.2 (N-type voltage-gated Ca^2+^ channel), SK (small conductance Ca^2+^ activated K^+^ channels; KCa2), HCN (hyperpolarization-activated cyclic nucleotide-gated cation channels), Kv4 (A-current; fast inactivating voltage-gated K^+^ channels, Kv4.2), and NaP (persistent Na^+^ current, Nav1.6; Maurice et al., 2004; Goldberg and Wilson, 2016). The AHP is determined mostly by SK channels (Bargas et al., 1999; Bennett et al., 2000; Goldberg and Wilson, 2005; Galarraga et al., 2007; Orduz et al., 2013). Voltage sag with hyperpolarizing current injection in ChIs is due to slow activation of HCN channels (Wilson, 2005). Deep resting membrane potentials are due to activation of inward rectifier K^+^ channels (Kir), and particularly in SPNs, the activation of Kir causes the characteristic deeper Vrest (Wilson, 2004; Shen et al., 2007). In two subtypes of SPNs, expression of Kir subtypes differs; Kir1 expression is observed in approximately half of iSPNs, while dSPNs express little Kir1, although most SPNs (both dPSNs and iSPNs) express Kir2 (Mermelstein et al., 1998). Kir1 shows inactivation and this inactivating Kir might contribute to slightly shallower Vrest in iSPNs compared with dSPNs (Mermelstein et al., 1998). SPNs exhibit a delay to the first action potential with depolarizing current injection (Kreitzer, 2009) due to fast- (Kv4.2) and slow-inactivating A-current (Kv1.2; Tkatch et al., 2000; Shen et al., 2004) and KCNQ2/3/5 channels (Kv7; Shen et al., 2005). Action potential width is determined mostly by repolarization rate. In FSIs, repolarization is mostly determined by Kv3.1 (Rudy and McBain, 2001), making typical fast-spiking narrow action potentials, while large conductance Ca^2+^-sensitive K^+^ channels (BK channels) determine repolarization in ChIs activated by Ca^2+^ influx through Cav2.1 (Goldberg and Wilson, 2005). In SPNs, action potential repolarization is also controlled by BK channels (Pineda et al., 1992).

Regional heterogeneity of ion channel expression in different subregions of the Str has not been studied extensively; our observations suggest possible regional heterogeneity of ion channel expression in particular cell types. Since membrane properties are determined by a combination of multiple types of ion channels, it is difficult to attribute differences of particular membrane properties to increase or decrease to a single ion channel type. For example, tonic spontaneous firing of ChIs can be modified in several ways. ChI tonic firing becomes faster and bursty with blockade of SK channels by apamin (Bennett et al., 2000). Blockade of Cav2.2 also makes ChI firing faster and bursty, since SK channels are coupled to Cav2.2 (Goldberg and Wilson, 2005). Reduced Nav1.6 current attenuates spontaneous firing of ChIs (Maurice et al., 2004). Therefore, increased spontaneous firing of ChIs could be attributed to reduced expression of SK or Cav2.2 or increased expression of Nav1.6. However, considering AHP amplitude in ChIs was substantially reduced by SK channel blockade (Bennett et al., 2000) and our observation of larger AHP amplitude and slow tonic firing rate of ChIs in the medial CPu, ChIs in the medial CPu might have higher SK channel expression than ChIs in other subregions. Since voltage sag in ChIs is mostly determined by HCN channels and ChIs express high levels of HCN1 and 3 (Muñoz-Manchado et al., 2018), another speculation is that the smaller sag in NAc shell ChIs reflects lower expression of HCN1 and 3.

Remarkably, the membrane properties of dSPNs and iSPNs differed more in the NAc than in the CPu. Since D1R and D2R colocalization in SPNs is more frequent in the NAc shell (~17%) than NAc core or CPu (~5%; Bertran-Gonzalez et al., 2008; Gangarossa et al., 2013), the opposite would have been expected if SPNs co-expressing D1R and D2R were included in the sampled neurons. However, D1R and D2R mouse lines only identify approximately two-thirds of the respective SPN cell populations (Shuen et al., 2008); SPNs co-expressing D1R and D2R might have been under-sampled due to weaker fluorescence labeling, accentuating differences between iSPNs and dSPNs. Our observations showed greater differences between dSPNs and iSPNs in the NAc; however, this does not mean dSPNs and iSPNs were the same in the CPu. Indeed, most of the parameters showed strong cell-type effects when only SPN subtypes were compared, while significant location and cell-type (SPN subtype) interactions were observed in only three parameters, indicating most of the parameters differed similarly between SPN subtypes regardless of location. The comparison suggests that the deeper Vrest and higher rheobase of dSPNs compared to iSPNs indicate that dSPNs are harder to excite, as previously shown (Kreitzer, 2009; Bergonzoni et al., 2021).

Although our observations of overall differences between dSPNs and iSPNs were comparable to previous studies, there were some discrepancies. One major reason is that most of the previous studies did not specify recording locations. In the current study, input–output curves did not differ significantly between dSPNs and iSPNs, while many reports showed input–output curves with more excitable iSPNs (Kreitzer and Malenka, 2007; Gertler et al., 2008). Although our observations did not show significant main cell type effects (overall dSPN-iSPN differences) in input–output curves, there were significant location and cell-type interactions, and dSPN and iSPN input–output curves were significantly different in the NAc shell and lateral CPu. This indicates that, if recording locations of previous studies were in the lateral CPu, our observations would not contradict the previous studies. Another factor to be considered is the age of mice. Most previous excitability studies in slices used younger mice (typical range is P14–P30) than our study (P60-93). Since membrane properties of SPNs change during postnatal development (Gertler et al., 2008), age differences cannot be ignored. Other possible factors are recording temperature and transgenic mouse strains. Ion channel open kinetics are temperature-sensitive (Hille, 2001); therefore active membrane properties recorded at room temperature (~22°C) and ~ 32°C may differ. Although we do not have evidence that mouse strain affects intrinsic membrane properties, it is possible that intrinsic cell properties show some differences in different transgenic lines.

In the present analysis, major neurotransmitter receptors were blocked to reveal regional heterogeneity in the intrinsic membrane properties of Str neurons. In slices, afferent inputs were cut and most Str neurons are not spontaneously active. However, there is spontaneous glutamate and GABA release, and transmitter receptors may be activated even without synaptic terminal stimulation. ChIs are spontaneously active in Str brain slices, and ACh acts at receptors expressed by most Str neurons, as well as both intra- and extra-striatal afferent synaptic terminals (Gonzales and Smith, 2015; Abudukeyoumu et al., 2019). Since spontaneous firing rate of ChIs shows regional heterogeneity, basal ACh tone may show regional variation, which is higher in the NAc shell and lower in the medial CPu. ACh receptor expression shows regional heterogeneity. For example, M1-type muscarinic ACh receptors (mAChR) are expressed in both subtypes of SPNs, and ACh increases excitability of SPNs by activating voltage-gated Ca^2+^ channels (Howe and Surmeier, 1995), suppressing KCNQ (Kv7) channel currents and Kir2 currents (Shen et al., 2005, 2007). M1 receptor-mediating Kir2 suppression is observed almost exclusively in dSPNs, while KCNQ suppression is similar in both subtypes of SPNs (Shen et al., 2007; Gertler et al., 2008). Because of possible heterogeneity in ACh tone, M1 receptor activation might be regionally heterogeneous and affect SPN excitability differentially. ChIs express M2/M4 mAChRs, activation of which suppresses activity of ChIs and release of ACh (Yan and Surmeier, 1996). This activation control of ChI involves different mACh subtypes in the NAc and CPu; M4 mediates the control in the NAc, while M2 and M4 do so in the CPu (Threlfell et al., 2010). Dopamine (DA) neuron axon terminals express nicotinic ACh receptors (nAChRs), and the activation of these nAChRs drives transmitter release from the terminals, with regional heterogeneity (Threlfell et al., 2012; Nelson et al., 2014; Yorgason et al., 2017; Kramer et al., 2022; Liu et al., 2022). DA neurons release at least three small molecular transmitters, and their synaptic connections in the Str show regional heterogeneity(Chuhma et al., 2023). These transmitters activate ion channels and may affect membrane properties in regionally different ways. Moreover, regional heterogeneity in membrane properties of Str neurons in brain slices will be greater in the absence of receptor blockade, even without stimulation. Previous studies varied in transmitter receptor blockade, which could be another source of discrepancy. As DA neuron axons are not spontaneously active in Str slices, we did not block DA receptors; however, we cannot rule out a contribution of spontaneous DA release to regional variation in membrane properties.

Future studies will need to address whether Str neuron properties differ in striosome and matrix compartments. Since the matrix comprises approximately 85% of the Str (Johnston et al., 1990), and the location of striosomes varies from animal to animal, our sample likely is biased to the matrix. Whether membrane properties are homogenous within regions or the regionality reflects gradients in membrane properties, as there are for gene expression (Gokce et al., 2016; Bengtsson Gonzales et al., 2020; Stanley et al., 2020), will require higher density sampling. Nonetheless, our study shows that intrinsic excitability properties are regionally heterogeneous in both Str projection neurons and interneurons, and the differences are mainly between the NAc and the CPu. Our study also highlights the uniqueness of the NAc shell in the membrane properties of all four cell types; NAc shell neurons are the easiest to excite, and dSPNs and iSPNs show differential responsivity.

Str subregions mediate different functions. The NAc receives excitatory input from limbic structure, e.g., the hippocampus, basolateral amygdala, and prefrontal cortex (Floresco, 2015). The NAc shell mediates reward-related cue signals and motivation, while the NAc core is involved in the selection of stimulus-appropriate response (Ikemoto, 2007; Floresco, 2015). The medial CPu receives inputs from association areas of the cortex and mediates action-outcome association and goal-directed action, while the lateral CPu receives inputs from primary motor and premotor cortices and mediates motor control and habit formation (Balleine et al., 2009). The most posterior part of the Str (‘tail’ of the Str) mediates novelty signals along with general salience signals (Menegas et al., 2017). The greater excitability of neurons suggests that smaller excitatory inputs can cause firing in these neurons, and differences in excitability can affect integration of synaptic inputs. Differential integration of synaptic input implies that intrinsic excitability differences may contribute to functional differences. The greater excitability of the NAc shell may enhance activity-dependent plasticity and contribute to the pivotal role of the NAc shell in reward-related learning and psychostimulant responsiveness (Klawonn and Malenka, 2018). Interestingly, intrinsic excitability of SPNs is modulated by conditioning, and modification of intrinsic excitability affects behaviors. Excitability of SPNs in the NAc shell, but not in the NAc core, is increased by fear conditioning (Zhang et al., 2023), and adolescent chronic intermittent ethanol exposure increases SPN excitability in both NAc shell and core but not in lateral CPu (Shan et al., 2019). For both of these results, the increased excitability is due to the reduction of the AHP by suppressing SK channel currents, and the injection of SK channel agonist in the NAc shell blocked ethanol-induced anxiety-like behaviors (Shan et al., 2019; Zhang et al., 2023). The greater excitability of NAc shell neurons may make the region more responsive to changed conditions, and the initiating point for long-term modification of Str circuits.

## Data availability statement

The datasets presented in this study can be found in online repositories (FigShare; DOI: 10.6084/m9.figshare.25229498; https://figshare.com/articles/dataset/Membrane_property_raw_data/25229498).

## Ethics statement

The animal study was approved by the IACUC, New York State Psychiatric Institute. The study was conducted in accordance with the local legislation and institutional requirements.

## Author contributions

NC: Conceptualization, Data curation, Formal analysis, Investigation, Methodology, Resources, Software, Validation, Visualization, Writing – original draft, Writing – review & editing. SR: Conceptualization, Funding acquisition, Project administration, Resources, Supervision, Visualization, Writing – original draft, Writing – review & editing.

## Acknowledgments

The authors thank Mihran Bakalian, Soo Jung Oh, and Gabriella Ortiz for their technical support and discussion.
