# Supplementary material for: Regional heterogeneity in the membrane properties of mouse striatal neurons
Source: Front Cell Neurosci. 2024 Jul 31;18:1412897. doi: 10.3389/fncel.2024.1412897 (PMC11321984; doi:10.3389/fncel.2024.1412897)
Supplement: Supplementary file 5 [file Data_Sheet_1.docx]

***Supplementary Material***

Regional heterogeneity in the membrane properties
of mouse striatal neurons

**Nao Chuhma*, Stephen Rayport***

*** Correspondence:** Nao Chuhma: [nao.chuma@nyspi.columbia.edu](mailto:nao.chuma@nyspi.columbia.edu), Stephen Rayport: stephen.rayport@nyspi.columbia.edu

# Supplementary Figures and Tables

Figure S1: Membrane property measurement schema (in this file)

Figure S2: PCA variable contribution (in this file)

Table S1: Parameter distribution in males and females in each cell type (see separate Excel file)

Table S2: Eigenvalues and variance explanation percentage of all PCs in each cell type (see separate Excel file)

Table S3: Post hoc comparison location/cell type interaction in Fig 2 (see separate Excel file)

Table S4: Post hoc comparison location/cell type interaction in Fig 3 (see separate Excel file)

Table S5: ANOVA results of SPN subtype comparison of input-output curve (in this file)


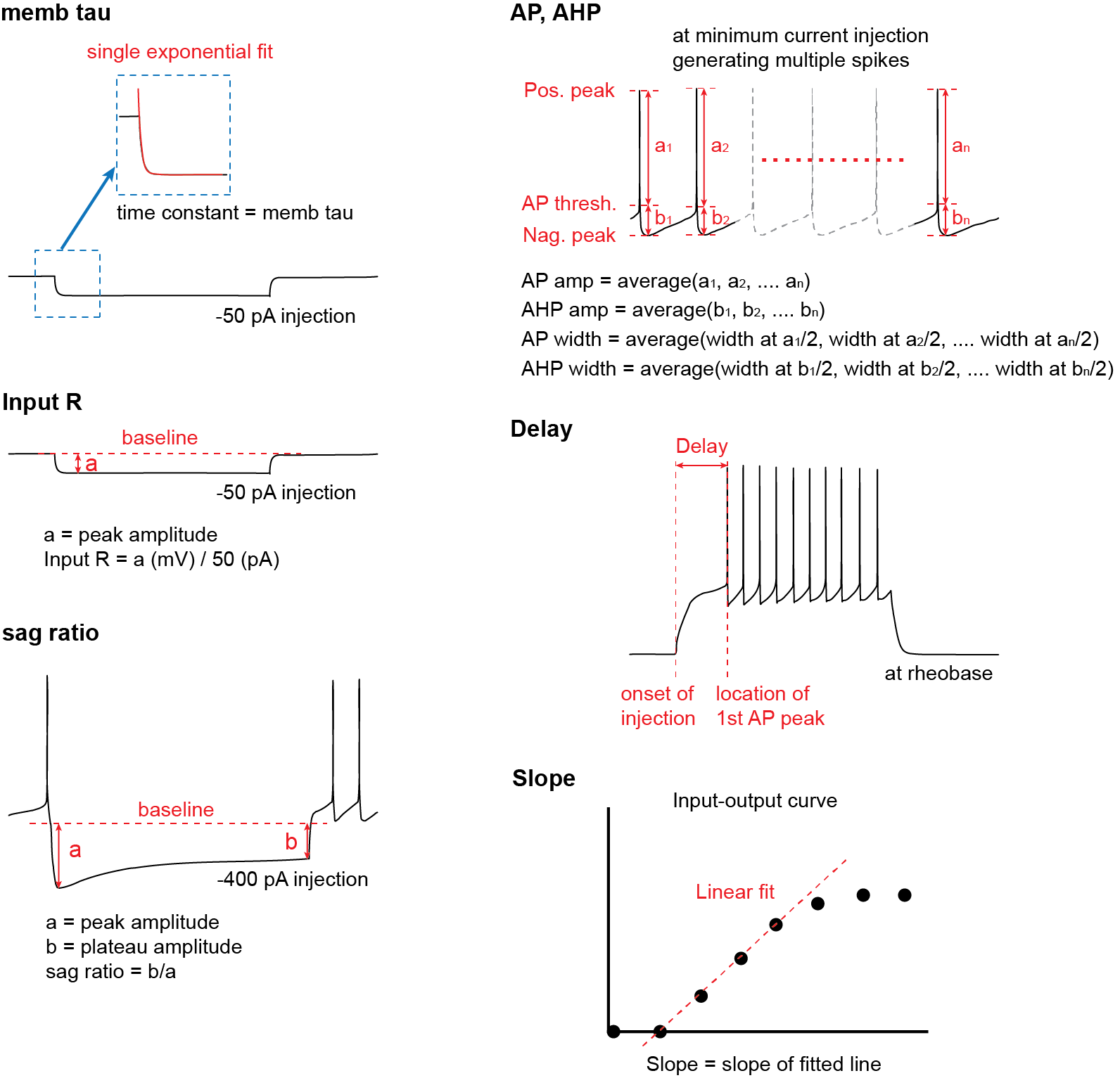


**Figure S1.** **Measurement of membrane properties.**

Schematics of membrane parameter measurements: membrane time constant (memb tau), input impedance (input R), sag ratio, action potential (AP) and after-hyperpolarization (AHP) amplitude (amp), width at 50% of peak amplitude, delay to the first AP, and input-output curve slope.


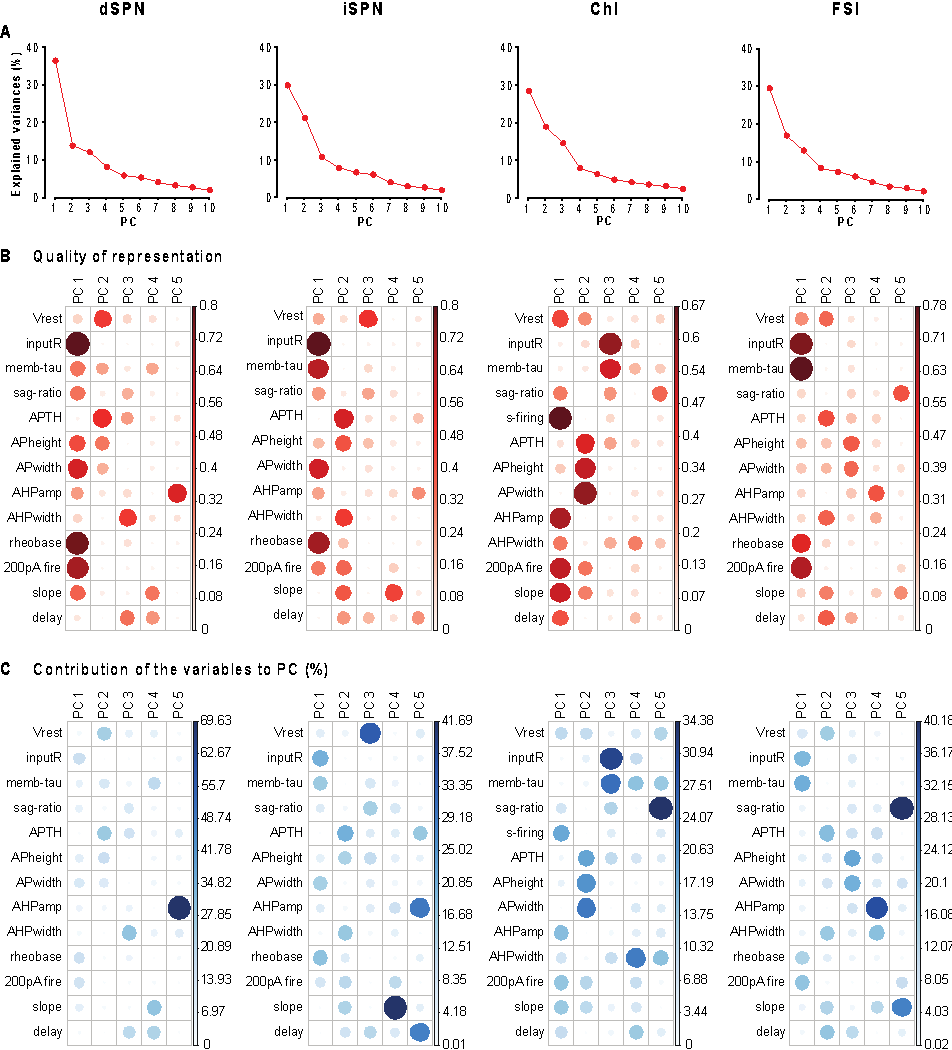


**Figure S2.** **Variables in PCA for each cell type**

**(A)** Scree plots of PC1 to PC10 in dSPN, iSPN, ChI, and FSI (from left to right), showing explained percentage of variation in the data by PC in the order of eigenvalues; the PC with the largest eigenvalue becomes PC1. For eigenvalue and actual number of explained percentage for all PCs, see **Table S2**. **(B)** Quality of representation of variables in PCs. Higher value (larger circle and darker red) indicates a good representation of the variables on the PC. Higher value corresponding to longer arrow along with the PC in PCA biplot (**Fig. 4**). Quality of representation was calculated as squared cosine (projection to the PC of ‘variable arrow’ in PCA biplot), therefore it has positive value regardless of direction. Perfect representation becomes 1. **(C)** Per cent contribution of variables to each PC. The contribution is calculated as quality of representation, a variable in a PC * 100 / total quality of representation of the PC. Variables that show a greater contribution to the PC are to the left (e.g. PC1), and are more important in explaining the variability of the data.

**Table S5. ANOVA results of SPN subtype comparison for input-output curves**

Results table of three-way mixed ANOVA for input-output curves (location, cell type, and current injection amount), and post hoc comparison of cell type (dSPN vs. iSPN) effect in each location. Df: degree of freedom, MSE: mean squared error, ges: general eta squared (effect size), estimate: estimated marginal means, SE: standard error. **, *** indicate p < 0.01, and p < 0.001, respectively.

**Main ANOVA table**

| **Effect** | **Df** | **MSE** | **F** | **ges** | **p.value** |
| --- | --- | --- | --- | --- | --- |
| **cell_type** | 1, 200 | 114.59 | 0.19 | <.001 | .666 |
| **location** | 4, 200 | 114.59 | 6.46 | .052 | <.001 *** |
| **cell_type : location** | 4, 200 | 114.59 | 4.47 | .036 | .002 ** |
| **injection** | 1.89, 377.38 | 82.84 | 149.87 | .302 | <.001 *** |
| **cell_type : injection** | 1.89, 377.38 | 82.84 | 0.19 | <.001 | .811 |
| **location : injection** | 7.55, 377.38 | 82.84 | 2.87 | .032 | .005 ** |
| **cell_type : location : injection** | 7.55, 377.38 | 82.84 | 4.03 | .044 | <.001 *** |

**Post hoc comparison, cell type / location interaction**

| **contrast** | **location** | **estimate** | **SE** | **Df** | **t.ratio** | **p.value** |
| --- | --- | --- | --- | --- | --- | --- |
| **dSPN - iSPN** | **shell_NAc** | 4.19 | 1.46 | 200 | 2.87 | 0.005** |
| **dSPN - iSPN** | **core_NAc** | 0.49 | 1.65 | 200 | 0.30 | 0.768 |
| **dSPN - iSPN** | **med_CPu** | -1.77 | 1.04 | 200 | -1.71 | 0.089 |
| **dSPN - iSPN** | **lat_CPu** | -3.21 | 1.11 | 200 | -2.89 | 0.004** |
| **dSPN - iSPN** | **post_CPu** | -0.98 | 1.28 | 200 | -0.77 | 0.444 |
